# Green teams drive sustainability: a European Rare Kidney Disease Reference Network Survey on dialysis environmental practices

**DOI:** 10.1093/ckj/sfaf276

**Published:** 2025-09-19

**Authors:** Emanuele De Simone, Marco Carmelo Morrone, Ece Izel Tatarhan, Marco Pozzato, Dario Roccatello, Roberta Fenoglio, Savino Sciascia

**Affiliations:** University Center of Excellence on Nephrologic, Rheumatologic and Rare Diseases (ERK-Net Member) with Nephrology and Dialysis Unit and Center of Immuno-Rheumatology and Rare Diseases (CMID), Coordinating Center of the Interregional Network for Rare Diseases of Piedmont and Aosta Valley, ASL Città di Torino, Turin, Italy; University Center of Excellence on Nephrologic, Rheumatologic and Rare Diseases (ERK-Net Member) with Nephrology and Dialysis Unit and Center of Immuno-Rheumatology and Rare Diseases (CMID), Coordinating Center of the Interregional Network for Rare Diseases of Piedmont and Aosta Valley, ASL Città di Torino, Turin, Italy; University of Turin. Department of Clinical and Biological Sciences, Turin, Italy; University Center of Excellence on Nephrologic, Rheumatologic and Rare Diseases (ERK-Net Member) with Nephrology and Dialysis Unit and Center of Immuno-Rheumatology and Rare Diseases (CMID), Coordinating Center of the Interregional Network for Rare Diseases of Piedmont and Aosta Valley, ASL Città di Torino, Turin, Italy; University Center of Excellence on Nephrologic, Rheumatologic and Rare Diseases (ERK-Net Member) with Nephrology and Dialysis Unit and Center of Immuno-Rheumatology and Rare Diseases (CMID), Coordinating Center of the Interregional Network for Rare Diseases of Piedmont and Aosta Valley, ASL Città di Torino, Turin, Italy; University of Turin. Department of Clinical and Biological Sciences, Turin, Italy; University Center of Excellence on Nephrologic, Rheumatologic and Rare Diseases (ERK-Net Member) with Nephrology and Dialysis Unit and Center of Immuno-Rheumatology and Rare Diseases (CMID), Coordinating Center of the Interregional Network for Rare Diseases of Piedmont and Aosta Valley, ASL Città di Torino, Turin, Italy; University of Turin. Department of Clinical and Biological Sciences, Turin, Italy; University Center of Excellence on Nephrologic, Rheumatologic and Rare Diseases (ERK-Net Member) with Nephrology and Dialysis Unit and Center of Immuno-Rheumatology and Rare Diseases (CMID), Coordinating Center of the Interregional Network for Rare Diseases of Piedmont and Aosta Valley, ASL Città di Torino, Turin, Italy; University of Turin. Department of Clinical and Biological Sciences, Turin, Italy

**Keywords:** chronic hemodialysis, dialysis, green nephrology, peritoneal dialysis, sustainable health

## Abstract

**Background:**

the environmental impact of healthcare is a growing concern, and nephrology, particularly dialysis, is a resource-intensive field. Dialysis involves high energy use, water consumption, significant waste generation, and transport-associated emissions. However, the extent to which “green” dialysis practices have been implemented remains largely unexplored.

**Methods:**

A comprehensive survey assessing the adoption of sustainable practices in dialysis covering water, energy, waste, transport and procurement policies was distributed through the European Rare Kidney Disease Reference Network (November 2024 to January 2025). The questionnaire covered key sustainability domains, including water and energy management, waste reduction, transport, and procurement policies. A “Green Score” was developed to quantify the implementation of eco-friendly initiatives. Additionally, a patient survey was conducted to evaluate perceptions of dialysis-related environmental impact and willingness to act.

**Results:**

A total of 34 nephrology centers (43% response rate) participated. The mean Green Score indicated that only 39.4% of achievable sustainability measures were in place. Despite widespread awareness, only 26.5% of centers had formal sustainability strategies, 17.6% had dedicated “Green Teams,” and just 12.1% utilized measurable indicators to track interventions. Water reuse systems were absent. Plastic recycling programs were present in 79.4% of centers, yet waste-saving initiatives were rare. Notably, the presence of a Green Team was significantly associated with higher Green Scores (*P* < .05). Patient responses (*n* = 45) revealed strong interest in sustainability, with 95.6% willing to take action.

**Conclusions:**

This study highlights critical gaps in sustainable dialysis practices across European nephrology centers. Despite interest, implementation remains limited. The strong association between Green Teams and sustainability scores highlights the need for formalized institutional efforts. Given the significant ecological footprint of dialysis, urgent action is required to integrate sustainable strategies into routine nephrology care.

KEY LEARNING POINTS **What was known:**

- Dialysis is resource-intensive, with significant environmental impact from energy, water, waste, and transport.
- While awareness of healthcare sustainability is growing, the adoption of “green” dialysis practices remains unclear.
- The extent of implementation of sustainable strategies in nephrology centers is largely unexplored.

**This study adds:**

- This European survey reveals a substantial gap between awareness and implementation of sustainable dialysis practices.
- Formal strategies, Green Teams, and measurable indicators are lacking in most centers.
- Despite patient willingness, widespread adoption of eco-friendly initiatives is poor, highlighting an urgent need for action.

**Potential impact:**

- These findings underscore the necessity of formalized institutional efforts, particularly the establishment of Green Teams, to drive the adoption of sustainable dialysis practices.
- The results advocate for urgent integration of sustainable strategies into routine nephrology care and policy development.

## INTRODUCTION

Sustainability has become a key concept across human activities, and healthcare practices are no exception, undergoing critical review in recent years [1]. The mandate is to foster sustainable approaches and discontinue activities incompatible with long-term ecological balance [2].

In the field of nephrology, a particular focus has been placed on dialysis procedures, which are recognized for their high energy consumption, substantial water usage, significant waste production, and the logistical demands of daily patient and material transport [3].

Recognizing the environmental impact of these practices, numerous nephrology centers worldwide have undertaken initiatives to mitigate their ecological footprint and promote environmentally sustainable (“green”) practices [4–6].

Several surveys have been conducted in recent years, particularly in Oceania, with the objective of establishing a foundational understanding of sustainable practices [7–9].

To comprehensively assess the current state of such initiatives across Europe and to determine the extent to which green practices have been adopted within nephrology centers, we conducted a survey distributed through the European Renal Disease Network (ERK-Net) [10]. ERK-Net is a European Reference Network connecting highly specialized healthcare providers to improve diagnosis and treatment of rare and complex kidney diseases across Europe through shared expertise, guidelines, and research. As members of this organization, we conducted this study with the aim to provide an in-depth analysis of the current landscape of sustainable practices in European nephrology, highlighting both the progress made and the areas requiring further attention, all within a homogeneous case study. We also decided to extend a specific survey to patients at our center to get their perspective on this topic. The cohort served by our home dialysis program was selected due to their direct engagement with critical aspects of environmental sustainability, including waste generation, water and energy consumption, and the use of disinfectants.

## MATERIALS AND METHODS

### Green nephrology survey

The green practices in dialysis survey consisted of a total of 24 questions. The survey was developed over the existing surveys and a first draft was sent to a European nephrology center (not affiliated with ERK-Net) for pilot testing to assess its clarity and burden. Following this pilot, five questions were added. Subsequently, three questions were excluded from the analysis due to potential misinterpretations. The survey was distributed among centers belonging to the ERK-Net between November 2024 and January 2025 through a Google form. The first part of the survey explored the different characteristics of the centers, specifically the country of origin, size, and type of patient treated. Subsequently, the general adoption of green policies within the center was considered. The four main items: water, waste, energy, and the transport and presence of indicators for the effectiveness of interventions were then explored. Finally, the last questions investigated the personal evaluation of the problem’s relevance. The survey is available in the Supplementary Materials.

#### Patient survey

The patient survey was administered to patients attending our center—San Giovanni Bosco Hub Hospital Turin—who underwent home dialysis, either peritoneal dialysis or hemodialysis, between November 2024 and January 2025, during a clinical visit. Our home dialysis program serves 48 patients: 34 in peritoneal dialysis and 14 in home hemodialysis.

The survey comprised a few questions (3 questions + 1 additional question for home hemodialysis patients) to investigate patients’ perceptions of the problem and their degree of engagement on this topic. The survey is available in the Supplementary Materials. The answers were collected anonymously.

#### Green Score

To provide a numerical reference to the responses received in the “Green Survey,” questions investigating the presence of specific eco-friendly practices implemented by the centers were compiled into a score called the “Green Score.” Each question was assigned a score from 1 to 5 based on its importance to green aspects, and the relative score was calculated for all centers based on whether they had already achieved that objective. The scoring was performed independently by two raters (one is an experienced nephrologist in the field of dialysis and the other professor of nephrology with Masters in sustainable health from the University of Turin), who subsequently compared their responses to assess inter-rater reliability.

The questions that contributed to the creation of this score and their corresponding point values are reported in the Supplementary Materials.

#### Statistical analysis

The two-variable statistical analysis was performed using the Student’s *t*-test, while the three-variable statistical analysis was performed using ANOVA. A two-sided *P*-value <.05 was statistically significant. All statistical analyses were performed using SPSS version 26.0 (IBM, Armonk, NY, USA).

## RESULTS

### Green dialysis survey

**Figure 1:**
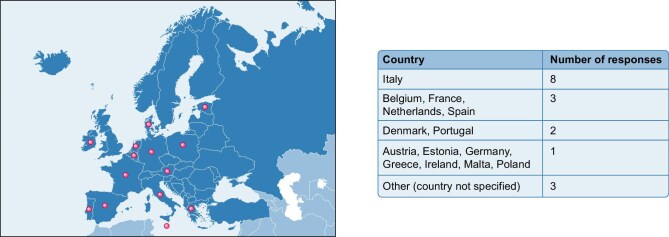
This map illustrates the geographical spread of responses, with green dots indicating the approximate location of contributing centers. The accompanying table provides the lists of countries from which responses were received and the number of responses received from each individual country. For rows listing multiple countries the number in this column signifies that each of those countries contributed that specific number of responses.

Total amount of responses received was 34. The response rate was 43% (34/79). Specifically, number of responses received for each country is summarized in Fig. 1.

Fourteen centers (41.2%) reported serving adult patients: 11 (32.3%) were pediatric centers, and nine centers (26.5%) had both units, pediatric and adult. Twenty-one centers (61.8%) accommodated fewer than 50 maintenance hemodialysis patients, 7 facilities (20.6%) had between 50 and 100 patients, 6 centers served >100 patients (17.6%). All the centers had a peritoneal dialysis program (100%), and 12 dialysis centers had a home hemodialysis program (36.4%).

**Figure 2:**
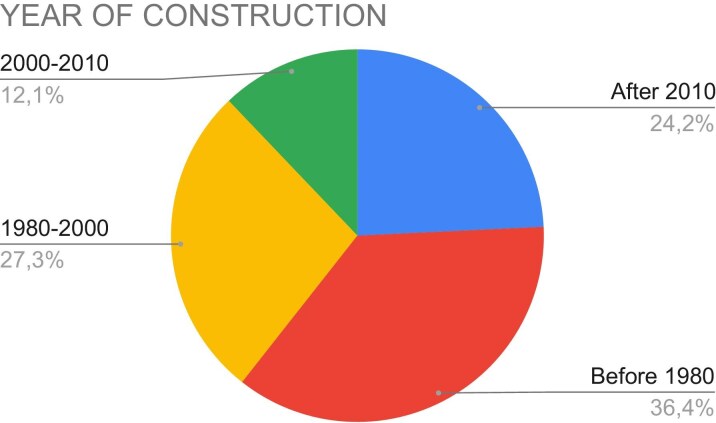
Percentage of construction year ranges of dialysis facilities.

More than 60% of the facilities were built before 2000 [12 facilities were built before 1980 (36.4%) and nine facilities between 1980 and 2000 (27.3%)], four facilities between 2000 and 2010 (12.1%), and eight facilities were built more recently, after 2010 (24.2%). One center did not answer to this question (Fig. 2).

### Green programs development

Nine centers (26.5%) reported implementing strategies or action plans for environmental protection. Qualitative data from open-ended responses revealed the following initiatives among the nine centers with plans: one had a dedicated Environmental Medicine Unit, one prioritized environmentally friendly materials and packaging, one had implemented a comprehensive digital transformation across all departments coupled with a system for evaluating air and electricity consumption and waste audits, and one center reported educational initiatives.

Regarding “Green Teams,” six centers (17.6%) reported having a formal team responsible for environmental initiatives. One additional center elaborated in an open response that while no formal Green Team existed specifically for dialysis, a physician and a nurse were focused on green initiatives. Among centers with Green Teams, three reported involvements in “waste reduction/separation and water/dialysate savings programs, reducing plastic consumption and use of disposable gloves.”

Eight centers (23.5%) had incorporated environmental criteria into their procurement contracts.

Twenty-one centers (61.8%) required information to assess the environmental impact of dialysis devices, equipment, and machines. Among the 21 centers requiring information, 11 sought recycling services from suppliers, four required packaging data, two requested information on the composition of uncontaminated materials, two requested information on the origin of dialysis machine components, and one requested both packaging data and supplier recycling services.

Data on green program development are summarized in Table 1.

**Table 1:** The answers to questions investigating the development of green programs.

Questions	Answer	Percentage, % (*N*)
Strategy or action plan for environmental protection	Yes	26.5 (9)
Formal Green Team	Yes	17.6 (6)
Greener solutions despite higher costs	Yes	88.2 (30)
Environmental criteria in procurement contracts	Yes	23.5 (8)
	I don’t know	26.5 (9)
Assess the environmental impact of dialysis devices, equipment and machines	Yes	61.8% (21)
Data on packaging	*N* = 5	14.7 (5)
Information on the composition of uncontaminated materials	*N* = 2	5.9 (2)
Information on the origin of dialysis machine components	*N* = 2	5.9 (2)
Availability of recycling services from suppliers	*N* = 12	35.3 (12)
Introduction of energy-saving strategy indicators	Yes	12.1 (4)

#### Water management

Thirty-two centers (94.1%) utsed reverse osmosis systems for dialysis water production. Fifteen centers (44.1%) employed a centralized dialysate preparation system. Wastewater reuse from reverse osmosis was not practiced at any center. Ten centers (29.4%) reported reducing dialysate flow for elderly or clinically appropriate patients to conserve water, while 21 (61.8%) did not. Three centers (8.8%) were unaware of this practice.

#### Energy management

Four centers (11.8%) used renewable energy sources: two solar, one wind, and one unspecified. Twenty-three centers (67.6%) did not use renewable energy. Ten centers (29.4%) implemented energy-saving programs, including: four centers using energy-saving light bulbs, four using automatic power shutdown, one using “reverse osmosis in stand-by” and one using reminder notes for personnel. Twenty centers (58.8%) did not have an energy-saving program.

#### Waste management

Thirty-one centers (91.2%) provided paper recycling bins in their facilities. Three centers quantified paper recycling: one estimated one bin per day, another reported 40% daily recycling, and one minimized paper use through electronic medical records. Three centers (8.8%) did not have paper recycling bins. Twenty-seven centers (79.4%) offered plastic recycling bins, with one center quantifying recycling at one bin per day. Seven centers (20.6%) did not have plastic recycling bins. Twenty-two centers (64.7%) provided glass recycling bins, but no center quantified glass recycling.

Seven centers (20.6%) implemented special waste-saving programs, but none provided details.

#### Transport

Twenty-eight dialysis facilities (82.4%) had on-site warehouses for material storage, while six (17.6%) did not. One center reported an off-site warehouse 32 km away. Twenty centers (58.8%) had telemedicine programs. Twenty-seven dialysis facilities (79.4%) promoted or provided patient travel options: 14 centers (41.2%) promoting public transportation, nine (26.5%) offering carpooling, and four (11.7%) encouraging walking or cycling. Two centers provided transportation via ambulance or similar services, one used a “Dialysis Taxi,” one tailored travel options to patient conditions.

Data for water, energy, and waste management, and carbon footprint and transport are summarized in Table 2.

**Table 2:** Answers to questions regarding the ecological footprint of the main items of dialysis.

Items and questions	Answer	Percentage, % (*N*)
**water**		
water production system		
reverse osmosis	yes	94.1 (32)
centralized preparation system	yes	44.1 (15)
reusage of wastewater	yes	0 (0)
dialysate flow reduction	yes	29.4 (10)
energy		
renewable energy	yes	11.8 (4)
energy-saving programs	yes	29.4 (10)
**waste**		
recycle bins		
paper	yes	91.2 (31)
plastic	yes	79.4 (27)
glass	yes	64.7 (22)
special waste-saving program	yes	20.6 (7)
transport		
on-site warehouse for storing dialysis materials	yes	82.4 (28)
telehealth	yes	58.8 (20)
travel options	yes	79.4 (27)

#### Indicators

Only four centers (12.1%) reported using measurable indicators to evaluate the adequacy of their environmental interventions. Specifically, one center calculated a potential water saving of 200 000 l through dialysate flow adjustment while maintaining dialysis efficacy. This center also reported a plastic waste separation capacity of 3000 l per week. One center investigated waste product elimination in patient-tailored treatments, demonstrating a reduction in waste dialysate.

#### Subjective relevance

Thirty participants (88.2%) indicated that they would opt for greener solutions despite potentially higher prices.

Participants rated the future importance of green dialysis on a scale of 0 to 100. The mean perceived importance was 82.3 (*n* = 32). Participants also rated the perceived preference of patients and their relatives for green dialysis strategies on a scale of 0 to 100. The mean perceived patient preference was 56.1 (*n* = 33), and the mean perceived relative preference was 54.8 (*n* = 33).

#### Green score

The mean Green Score was 16.1 out of a maximum of 41 points, representing a green practices application rate of 39.4%. A statistically significant correlation was found between the presence of a formal Green Team and the Green Score (*P* < .05). The Green Score of the centers was compared according to factors including center size and type. No statistically significant correlations were observed between the Green Score and these factors.

### Patient survey

#### Home hemodialysis

Fourteen patients on home hemodialysis responded to the survey (100%). Eight patients (57.1%) used the NxStage system with a 4–5 sessions/week schedule, and six patients (42.9%) used the Nikkiso machine with a 3.5 sessions/week schedule. Regarding the environmental impact of their treatment, seven patients (50%) considered it “very impactful,” two (14.3%) “moderately impactful,” one (7.1%) “slightly impactful,” and four (28.6%) had not considered the issue. All 14 patients (100%) reported proper disposal of special and domestic waste from their dialysis sessions. Eight patients (57.1%) prioritized environmental concerns and were willing to take further action to reduce their treatment’s impact, while six patients (42.9%) were willing to do more within the limits of their health.

#### Peritoneal dialysis

Thirty-one patients on peritoneal dialysis responded to the survey (91%). Twenty patients (64.5%) were on continuous ambulatory peritoneal dialysis with 1–4 daily exchanges, and 11 patients (35.5%) used automated peritoneal dialysis. Regarding environmental impact, 11 patients (35.5%) considered it “very impactful,” 14 (45.2%) “moderately impactful,” one (3.2%) “slightly impactful,” and five (16.1%) had not considered the issue. Twenty-nine patients (93.5%) reported proper waste disposal, and two (6.5%) reported partial disposal. Seventeen patients (54.8%) prioritized environmental concerns and were willing to take further action, 12 (38.7%) were willing to do more within health limits, and two (6.5%) were not willing to do more.

## DISCUSSION

This international study provides an overview of environmental sustainability practices in dialysis facilities across Europe, focusing on the relatively homogeneous network of centers within the European Rare Kidney Disease Reference Network: ERK-Net. The survey achieved a 43% response rate. Comparing these findings to similar surveys, an Australian study conducted in the state of Victoria reported a high response rate of 86% [7]. However, when expanded to include both Australia and New Zealand, the response rate dropped to 33%, aligning more closely with our results [8]. Similarly, the Portuguese National Survey reported a response rate of 26% [9].

Most participating centers were small, with ∼60% serving <50 maintenance hemodialysis patients. Notably, >60% of the facilities were established before 2000, suggesting that many operate within aging infrastructure.

Only six centers (17.6%) had a formal Green Team dedicated to environmental initiatives, with their efforts primarily focused on waste management. When assessing sustainability practices using the Green Score, we found that, on average, centers implemented only 39.4% of the total achievable sustainability measures. Importantly, our analysis identified a statistically significant correlation between the presence of a Green Team and a higher Green Score, suggesting that such teams play a key role in advancing sustainability within dialysis facilities. To our knowledge, this is the first study to demonstrate this association. Given these findings, nephrology societies and healthcare policymakers should prioritize the establishment of formal Green Teams in dialysis centers as a critical step toward integrating sustainable practices into routine care.

Despite growing awareness of sustainability, concrete actions remain limited. Only nine centers had implemented a formal action plan for environmental sustainability, and just eight (23.5%) incorporated green criteria into their procurement contracts. Regarding this last aspect, it is noteworthy how low the figure remains, despite nearly 90% of respondents expressing that green solutions should be pursued, even at the expense of higher costs. This observation underscores the complexity inherent in transitioning from individual willingness to the establishment of organized and validated institutional models. Furthermore, for centers that had integrated these criteria into their tender specifications, no additional details regarding the implementation process were available. We contend that the inclusion of such criteria in tenders is paramount for securing industry support in this area. It is therefore highly desirable that centers accumulating expertise in this sector disseminate their experiences to foster the rapid advancement of this theme, thereby assisting other centers in precluding instances of mere “greenwashing.”

Notably, no center reported reusing wastewater from reverse osmosis systems, despite documented strategies for such reuse in the literature [11] and a 2017 survey in Australia and New Zealand reporting a 13% adoption rate [8]. Furthermore, only 10 centers (29.4%) adjusted dialysate flow based on patient needs, even though evidence suggests this practice does not compromise clinical outcomes in selected cases [12]. Encouraging its broader adoption could represent a simple yet effective conservation strategy, which is just one among many measures that can be implemented in this and other areas of dialysis [13].

Waste management efforts varied. While paper recycling was widely implemented, plastic and glass recycling were less consistent, despite plastic being the predominant waste product in dialysis facilities. A single dialysis session generates between 1.5 and 8 kg of waste, with proper management significantly impacting overall environmental burden [14]. Only 15 centers (44.1%) used centralized dialysate production: a practice that can reduce plastic bag consumption and water use, given that ∼180 l of water are required to produce 1 kg of plastic [6]. While implementing centralized dialysate systems may require initial investment, it is often cost-saving in the medium term and should be promoted through formal support from healthcare authorities and governments. Additionally, special waste management remains an area of concern; only seven centers (20.6%) reported having programs to reduce special waste, and none provided specific implementation details.

Energy sustainability efforts were similarly limited. Only four centers (12%) reported using renewable energy sources, a figure consistent with the 14% reported in the 2017 survey by Talbot and colleagues [8]. Only 10 centers (29.4%) had established energy-saving programs.

Regarding transportation, nearly 80% of centers promoted or facilitated travel options aimed at reducing carbon emissions. However, telemedicine was underused, with only 20 centers (58.8%) reporting active programs. Nevertheless, it should be noted that our survey was not designed to quantify the reduction in in-person visits attributable to telemedicine programs, nor the decrease in material movement facilitated by in-house storage.

A critical gap identified in this study was the lack of measurable indicators—such as water or electricity meters—to assess the effectiveness of sustainability interventions. Only four centers (12.1%) reported using such metrics, as reported in the results. However, in some cases, centers presented indicators for only a single item rather than an cohesive strategy, possibly also resulting from extemporaneous re-evaluations rather than a longitudinal approach. It is desirable that centers equip themselves with indicators across various areas of environmental sustainability that are enduring over time, allowing for the evaluation of green performance. The development and sharing of these indicators would enhance comparability and the self-assessment necessary for planning any further interventions.

Our survey also explored perceptions of dialysis-related environmental impact. While healthcare professionals acknowledged the importance of sustainability and expressed a willingness to adopt greener solutions despite potential cost increases, they perceived lower interest from patients. However, this contrasts with our center’s experience, where 75.6% of patients demonstrated substantial awareness of dialysis-related environmental concerns. Furthermore, 95.6% of patients surveyed expressed a willingness to take further action to minimize the environmental footprint of their treatment. These findings align with the principles of “patient empowerment and self-care” as key components of sustainable healthcare [15].

This study has several limitations. The findings from 34 centers may not be fully generalizable to the broader European dialysis landscape, particularly given a disproportionately higher response rate from certain countries, notably Italy. The survey was designed to be simple and minimally burdensome, potentially limiting the depth of collected data, and there was no requirement for the respondent to be the most qualified to answer. Furthermore, selection bias is a potential limitation. Responses may have been skewed toward centers that are already more committed to environmental sustainability, and the ERK-Net community might not accurately reflect practices in other centers within the same countries. Another limitation inherent in self-assessment within this area is the potential for social desirability bias. Moreover, the intrinsic nature of the Green Score inherently limits it to the subjective assessments made by the raters. Some questions—particularly those related to perception—relied on subjective responses, which may introduce variability. Finally, we did not address the perception of in-center patients, whose treatment is often beyond their control, and who also need to be empowered.

In conclusion, this study highlights several critical gaps in the sustainability practices of European dialysis centers. Key areas for improvement include water conservation, plastic waste reduction, and the expansion of telemedicine. Additionally, the adoption of measurable sustainability indicators remains rare, and only a minority of centers have established Green Teams, despite the strong correlation between Green Teams and higher Green Scores identified in our analysis. Finally, patients represent an often-overlooked yet motivated stakeholder group in sustainability efforts. Patients can be actively involved in the center’s green initiatives, not least because knowing their therapies are less polluting or more sustainable than current practices can be a source of relief for many. A shift toward more structured environmental initiatives is urgently needed, requiring commitment from nephrology societies, healthcare policymakers, and all involved stakeholders. Sustainable dialysis is not merely an option but a necessity: for ourselves, future generations, and the planet.

## Supplementary Material

sfaf276_Supplemental_File

## Data Availability

Data are available upon reasonable request.
