# Supplementary material for: Green teams drive sustainability: a European Rare Kidney Disease Reference Network Survey on dialysis environmental practices
Source: Clin Kidney J. 2025 Sep 19;18(10):sfaf276. doi: 10.1093/ckj/sfaf276 (PMC12541384; doi:10.1093/ckj/sfaf276)
Supplement: sfaf276_Supplemental_File [file sfaf276_supplemental_file.pdf]

SUPPLEMENTARY MATERIALS

Index:

1. Green nephrology survey
2. Patients survey – home hemodialysis
3. Patients survey - peritoneal dialysis
4. Green score questions

1- GREEN NEPHROLOGY SURVEY

**INTRODUCTION**	
**1. Please indicate your dialysis center:**	(Open text response)
**2. What type of patients does your dialysis center serve?**	- Pediatric<br>- Adult<br>- Both
**3. How many maintenance hemodialysis patients are currently at your center?**	- Less than 50<br>- Between 50 and 100<br>- More than 100
**4. Does your dialysis center have the following programs?**	
Home Hemodialysis	- *Yes*<br>- *No*
Peritoneal Dialysis	- *Yes*<br>- *No*
***5. Does your center have a Kidney Transplant Program?**	- Yes<br>- No
**6. When was the hospital facility or your dialysis center built?**	- Before 1980<br>- 1980-2000<br>- 2001-2010<br>- After 2010
**7. Does your center have a strategy or action plan for environmental protection, such as introductory trainings or workshops?**	- Yes<br>- No

If yes, please provide details:	*(Open text response)*
**8. Is there a formal "Green Team" responsible for environmental initiatives within your dialysis center or facility?**	- Yes<br>- No<br>- I don’t know
If yes, has it implemented any changes? Please detail:	*(Open text response)*
***9. Are you a decision maker when it comes to choice of type of dialysis?**	- Yes<br>- No<br>- Not yet
**10. If you were a decision maker, would you opt for greener solutions despite potentially higher prices?**	- Yes<br>- No
**11. Have you introduced environmental criteria in your procurement contracts?**	- Yes<br>- No<br>- I don’t know
**12. Do you require information to assess the environmental impact of dialysis devices, equipment, and machines?**	- Yes<br>- No<br>- I don’t know
If yes, please select all that apply:	*- Data on packaging*<br>*- Information on the composition of uncontaminated materials*<br>*- Information on the origin of dialysis machine components*<br>*- Availability of recycling services from suppliers*
**WATER MANAGEMENT**	
**13. Which kind of dialysis water production system do you use?**	- Reverse osmosis<br>- Centralized water preparation system

	- I don't know<br><br>- Others (please detail):<br><br>____________________
**14. Is wastewater reused for different purposes?**	- Yes<br><br>- No<br><br>- I don't know
If yes, please select all that apply:	*- Toilet*<br><br>*- Janitor station*<br><br>*- Gardens*<br><br>*- Other (please detail):*<br><br>____________________
**15. Do you use dialysate flow reduction for elderly patients or those with clinical conditions that allow it as a strategy to reduce water consumption?**	- Yes<br><br>- No<br><br>- I don't know
***16. Have you ever used Sorbent Dialysis at your facility?**	- Yes<br><br>- No<br><br>- Not yet<br><br>- I don't know
**ENERGY MANAGEMENT**	
**17. Do you use renewable energy to power your dialysis center?**	- Yes<br><br>- No<br><br>- I don't know
If yes, please specify the source(s):	*- Water*<br><br>*- Wind*

	- Solar<br>*- Other (please detail):*<br>____________________
**18. Have you implemented an energy-saving program at your dialysis center (e.g. energy-saving light bulbs, automatic power shutdown of electrical equipment)?**	- Yes<br>- No<br>- I don't know
If yes, please detail:	*(Open text response)*
**WASTE MANAGEMENT**	
**19. Are there recycling bins in your dialysis center for the following materials?**	
<u>Paper</u>	*- Yes*<br>*- No*
If yes, can you quantify the amount recycled?	*(Open text response)*
<u>Plastic</u>	*- Yes*<br>*- No*
If yes, can you quantify the amount recycled?	*(Open text response)*
<u>Glass</u>	*- Yes*<br>*- No*
If yes, can you quantify the amount recycled?	*(Open text response)*
**20. Special waste: have you implemented a special waste saving program at your facility?**	- Yes<br>- No<br>- I don't know
If yes, please detail:	*(Open text response)*
**CARBON FOOTPRINT AND TRANSPORT**	

**21. Do you have an on-site warehouse for storing dialysis materials?**	- Yes<br>- No
If no, how far away is the nearest warehouse (in kilometers)?	*(Numeric response)*
**22. Do you use Telehealth services?**	- Yes<br>- No<br>- Not yet
**23. Which of the following travel options are promoted or provided to patients? (Please select all that apply):**	- Encouragement to walk or cycle<br>- Use of public transportation<br>- Carpooling programs<br>- Other (please detail): ____________
**CONCLUSION**	
**24. Referring to the energy-saving strategies described in the paragraphs above (water, energy management, waste management), have you used any measurable indicators to confirm the strategy improved?**	- Yes<br>- No<br>- I don't know
If yes, please provide detail:	*(Open text response)*
**25. How important do you believe that green dialysis will be in the future (0-100)?**	*(Numeric response)*
**26. On a scale from 0-100, how important do you believe that patients would prefer a green dialysis strategy?**	*(Numeric response)*
**27. On a scale from 0-100, how important do you believe that patients families would prefer a green dialysis strategy?**	*(Numeric response)*

* These 3 questions were excluded from the analysis after the survey was conducted due to possible misinterpretations.

2- *PATIENTS SURVEY – Home Hemodialysis*

1) **How do you judge the environmental impact (in terms of energy consumption, water consumption and waste production) of your dialysis treatment?**
- **Very impactful**<br>- **Moderately impactful**<br>- **Slightly impactful**<br>- **I have never considered the problem**
2) **At home, are you careful to put only and exclusively special waste in the special waste bin?**
- **Yes, always**<br>- **no**<br>- **partially**
3) ***If the answer to the previous question is yes,*** **do you dispose of the remaining dialysis waste (e.g. paper or plastic) in the appropriate household recycling containers?**
- **Yes, always**<br>- **no**<br>- **partially**
4) **If in the future it were possible to further reduce the environmental impact of your treatment, would you be willing to increase your commitment to it?**
- **yes, environment is my priority**<br>- **I would be willing to do something more, compatibly with my health problems**<br>- **I am not willing to do more than I already do**

3- PATIENTS SURVEY – Peritoneal dialysis

1) **How do you judge the environmental mpact (in terms of energy consumption, water consumption and waste production) of your dialysis treatment?**
- **Very impactful**<br>- **Moderately impactful**<br>- **Slightly impactful**<br>- **I have never considered the problem**
2) **At home, do you properly dispose of dialysis waste (separating unsorted waste from what could be recycled)?**
- **Yes, always**<br>- **no**<br>- **partially**
3) **If in the future it were possible to further reduce the environmental impact of your treatment, would you be willing to increase your commitment to it?**
- **yes, environment is my priority**<br>- **I would be willing to do something more, compatibly with my health problems**<br>- **I am not willing to do more than I already do**

4- Green score – questions and scores

QUESTIONS	POINTS
7. Does your center have a strategy or action plan for environmental protection, such as introductory trainings or workshops?	**5**
11. Have you introduced environmental criteria in your procurement contracts?	**5**
12. Do you require information to assess the environmental impact of dialysis devices, equipment, and machines?	**3**
13. Which kind of dialysis water production system do you use?	**1 or 2***
14. Is wastewater reused for different purposes?	**2**
15. Do you use dialysate flow reduction for elderly patients or those with clinical conditions that allow it as a strategy to reduce water consumption?	**4**
17. Do you use renewable energy to power your dialysis center?	**4**
18. Have you implemented an energy-saving program at your dialysis center (e.g. energy-saving light bulbs, automatic power shutdown of electrical equipment)?	**2**
19 a. Are there paper recycling bins in your dialysis unit?	**1**
19 b. Are there plastic recycling bins in your dialysis unit?	**1**
19 c. Are there glass recycling bins in your dialysis unit?	**1**
20. Special waste: have you implemented a special waste saving program at your facility?	**4**
22. Do you use Telehealth services?	**4**
23. Which of the following travel options are promoted or provided to patients?	**3***
**TOTAL POINTS**	**41**

*For question number 13: if the response was “Reverse osmosis” 1 point, if the response was “Centralized water preparation system” 2 points.

*For question number 23: 3 points awarded for the answer “Encouragement to walk or cycle” or “Use of public transportation” or “Carpooling programs”.
